# Changes in lung immune cell infiltrates after electric field treatment in mice

**DOI:** 10.1038/s41598-021-81174-y

**Published:** 2021-01-14

**Authors:** Sophia I. Eliseeva, Zackery A. Knowlden, Gillian MSchiralli Lester, David A. Dean, Steve N. Georas, Timothy J. Chapman

**Affiliations:** 1 Division of Pulmonary and Critical Care Medicine, Department of Medicine, University of Rochester Medical Center, Rochester, NY USA; 2 Departments of Pediatrics, Biomedical Engineering, and Pharmacology and Physiology, University of Rochester Medical Center, Rochester, NY USA; 3 Center for Infectious Diseases and Immunity, Rochester General Hospital Research Institute, Rochester Regional Health, 1425 Portland Ave, Room 403, Rochester, NY 14621 USA

**Keywords:** Cell biology, Immunology, Respiratory tract diseases

## Abstract

Exogenous electric fields are currently used in human therapy in a number of contexts. Interestingly, electric fields have also been shown to alter migration and function of immune cells, suggesting the potential for electric field-based immune therapy. Little is known as to the effect of electric field treatment (EFT) on the lung. To determine if EFT associates with changes in lung immune cell infiltration, we used a mouse model with varying methods of EFT application and measured cells and soluble mediators using flow cytometry and cytokine/chemokine multiplex. EFT was associated with a transient increase in lung neutrophils and decrease in eosinophils in naïve mice within 2 h of treatment, accompanied by an increase in IL-6 levels. In order to test whether EFT could alter eosinophil/neutrophil recruitment in a relevant disease model, a mouse model of allergic airway inflammation was used. Four EFT doses in allergen-sensitized mice resulted in increased neutrophil and reduced eosinophil infiltrates following allergen challenge, suggesting a durable change in inflammation by EFT. Mice with allergic inflammation were analyzed by flexiVent for measures of lung function. EFT-treated mice had increased inspiratory capacity and other measures of lung function were not diminished. These data suggest EFT may be used to manipulate immune cell infiltration in the lung without affecting lung function.

## Introduction

Normal cellular physiology involves the generation of endogenous electric fields across membranes and body sites that promote movement of cells and solutes to maintain homeostasis. Recently, the use of exogenous electric fields for the purpose of cell or tissue therapy has become an area of interest. For research purposes, electroporation is a key technology that enables the transfer of genetic material into cells via transfection by increasing electric potential across a cell membrane, thereby creating membrane pores^1,2^. Cells of different size require different voltage to permeabilize, therefore allowing for size-based selection of cells for transfection in a mixed culture^3,4^. In clinical practice, muscle stimulation, cardiac function, and wound healing-based electric field therapies have all shown benefit and are areas of ongoing research^5^. Exogenous electric fields have also been used to manipulate immune cells. DNA vaccines have been shown to be more immunogenic in the skin when using exogenous electric field at electroporation-level voltage, likely due to local cell death and mobilization of dendritic cells and macrophages^6^. Changes in membrane polarization can also affect cytokine receptor membrane mobility and downstream intracellular signaling^7^. Of particular therapeutic interest, exogenous electric field treatment (EFT) of tumors was shown to induce improved anti-tumor immunity by increasing the number of tumor-infiltrating lymphocytes^8^. Additionally, EFT could prevent metastases after therapy in animal models following elimination of primary tumor, implying induction of long-lasting immunity^8–10^. These studies suggest that manipulation of immune cells via EFT may be employed to alter or improve inflammatory disease using non-invasive methods.

Despite the potential promise of EFT for various applications, little is known as to the effect of exogenous electric fields on the airways and lung function, and whether there may be therapeutic benefit in diseases such as allergic asthma. Asthma is a common disease characterized by inflammation of the bronchi and airway narrowing due to inappropriate immune reactions to inhaled allergens. Eosinophilic asthma is a major health burden worldwide with incidence up to 20% in some geographic locations and age groups^11^. In susceptible individuals, inhaled allergens deposit in the respiratory tract and lead to eosinophil-dominant inflammation. Therapies for eosinophilic asthma include inhaled and systemic corticosteroids, as well as new targeted monoclonal antibodies that suppress mucosal inflammation^12^. These therapies are targeted at symptoms and are limited in efficacy for modifying the disease process. EFT could potentially affect the disease process by modifying immune infiltrates or cytokines and chemokines produced in response to allergens.

We sought to determine the impact of EFT on pulmonary inflammation and lung function by using total lung application of electric field with a range of dosing and timing parameters. In addition, EFT was applied in a mouse model of allergic inflammation to determine the effect on Th2-driven upper airway inflammation. We found EFT-induced changes in the lung immune response that may be further developed for future therapies.

## Methods

### Mice and preparation of samples

C57Bl/6 mice from NCI were used for all studies. Animals were housed in the vivarium at the University of Rochester Medical Center in specific pathogen-free conditions. All experimental procedures were performed in accordance with institutional guidelines for the care and use of laboratory animals in an AALAC-approved facility. All animal protocols were approved by the University of Rochester Institutional Animal Care and Use Committee. *Preparation of lung samples*. For immunohistochemistry, lungs were inflated with formalin, excised and embedded in paraffin. Lung tissue was cut into five micron sections, deparaffinized, steamed at pH 9.5 for antigen retrieval and blocked with goat serum in addition to Streptavidin/Biotin blocking reagents (Streptavidin/Biotin blocking kit #SP-2002 Vector Labs, Burlingame, CA.). Tissue sections were incubated overnight at 4 °C with 1ug/ml anti-Neutrophil antibody (NIMP-R14 #ab2557; AbCam, Cambridge, MA.) followed by a goat anti-rat biotinylated secondary antibody at 7.5 μg/ml for 30mins (Vector Labs, #BA9400). The tissue sections were labeled using Vectastain Avidin/Biotinylated Complex ABC kit and then reacted with DAB Peroxidase substrate (Vector Labs, #SK-4100). For multiplex and flow cytometry analyses, lungs were excised and homogenized in 1 mL PBS over a tea strainer. After centrifugation, supernatant was collected in labelled tubes for multiplex analysis. Lung pellets were then incubated at 37 °C in a solution of 0.1 mg/mL DNase I (Sigma-Aldrich, #DN25) and 0.1 mg/mL Liberase (Roche, #5401119001) for 40 min. Lungs were periodically shaken to ensure even digestion of tissue. Lungs were then filtered through a 100um strainer and centrifuged. Supernatant was discarded and red blood cells lysed. Cells were again filtered through a 100um strainer, centrifuged, and re-suspended in 300uL of PBS for flow cytometry analysis. *Preparation of spleen samples*. Spleens were excised into 5 ml complete media and glass homogenizers used to break open tissue. Resulting homogenate was filtered through a 100um strainer and centrifuged. Red blood cells were lysed, and samples was filtered again prior to centrifugation. Cells were re-suspended in PBS for flow cytometry analysis.

### Neutrophil in vitro cultures

EFT-treated or control lungs were digested as above, then single-cell suspensions were subjected to negative enrichment using the MACS neutrophil isolation kit (Miltenyi). Resulting cell population from separate samples was plated in complete media at 3 × 10^5^ cells per well in a 96-well round bottom plate in the presence or absence of 5 μM R848 (Invivogen). Neutrophils alone were harvested at 4 h to detect spontaneous IL-6 release, while R848-stimulated neutrophils were harvested after 16 h incubation. Supernatants were tested for IL-6 levels by ELISA (Invitrogen).

### Electric field treatment

Mice were exposed to 4% isoflurane via nose cone in a biosafety cabinet. Once anesthetized, pad or tweezer electrodes were coated with conducting gel to avoid direct animal contact and applied across the chest wall (pad electrodes) or across the nasal bridge (tweezer electrodes, BTX, Harvard Apparatus). In order to generate exogenous electric fields for EFT, a square-wave electroporator was used that delivers a set voltage for a defined amount of time (ECM 830 Square Wave Electroporator, Harvard Apparatus). Standard EFT dosing was eight 10-ms pulses at 200 V/cm. The distance between the electrodes was calculated to account for variability in delivery between nasal bridge and chest wall methods. After EFT, conducting gel was cleaned off the fur and animals were returned to cages and monitored for recovery. Mock EFT involved isoflurane exposure and application of conducting gel in the same way, but in the absence of EFT. Initial studies were performed with a range of pulse lengths and doses that resulted in the approach used for the presented studies (data not shown).

### Allergic inflammation model

Naïve mice were sensitized to chicken ovalbumin (OVA; Sigma grade V) by intraperitoneal injection of 20ug in 4 mg aluminum hydroxide on day 0 and 100ug OVA in 5 mg aluminum hydroxide on day 6. From day 8 to 13, six consecutive days of OVA challenge were done by intranasal administration of 10ug OVA in 20uL PBS. Two days after final challenge, mice were sacrificed for endpoint analyses.

### Flow cytometry and multiplex

Cytokines and chemokines were measured from lung homogenate using custom multiplex assays (Bio-Rad) per manufacturer’s instructions. Assays were run on a Bio-Rad Bio-Plex instrument at the University of Rochester Human Immunology Core, and flow cytometry was performed in the University of Rochester Flow Core. Lung single-cell suspensions were pre-stained with Fc block and then stained with fluorochrome-conjugated antibodies. After washing, cells were re-suspended in PBS and analyzed using a custom configuration LSRII (BD). For all studies, the following antibody panel was used: F4/80 (FITC), Siglec-F (PE), CD45 (PE-Dazzle 594), B220 (PE/Cy5), Ly6G (PE/Cy7), CD64 (APC), CD103 (APC-R700), CD11b (APC-Fire 750), Live/Dead (Aqua/Pacific Orange), MHC Class II I^a^/I^e^ (Pacific Blue), and CD11c (Brilliant Violet 605). All antibodies and Live/Dead stain were from Biolegend. Appropriate negative and fluorescence minus one controls were used for all experiments. After acquisition, analysis was done using FlowJo software version 10 (BD).

### Measurement of lung function

Adult mice were anesthetized with ketamine/xylazine (100 mg/kg), tracheostomized via a 19-gauge blunt tip cannula, and placed on a small mammal ventilator (flexiVent, SCIREQ, Montreal, Canada). Animals were kept warm using a heating pad and monitored via EKG. Once the EKG leads were in place, mice were paralyzed with 1 mg/kg Vencuronium bromide to ensure they made no spontaneous movement during experimentation. Mice were mechanically ventilated with a tidal volume of 10 mL/kg, 150 breaths/min, PEEP of 3 cm H_2_O, and FIO2 of 21–50%. Mice were challenged with increasing doses of methacholine (0–100 mg/mL in sterile PBS) with several ventilation maneuvers to characterize the airways. After the last methacholine dose, mice were sacrificed by ketamine overdose and lungs were collected for further analysis.

### Statistics

All group comparisons were done using one-way ANOVA with Tukey’s multiple comparisons test. For lung function measurements, groups were compared across the methacholine dose range using two-way ANOVA. A *p* value < 0.05 was considered significant.

## Results

**Figure 1:**
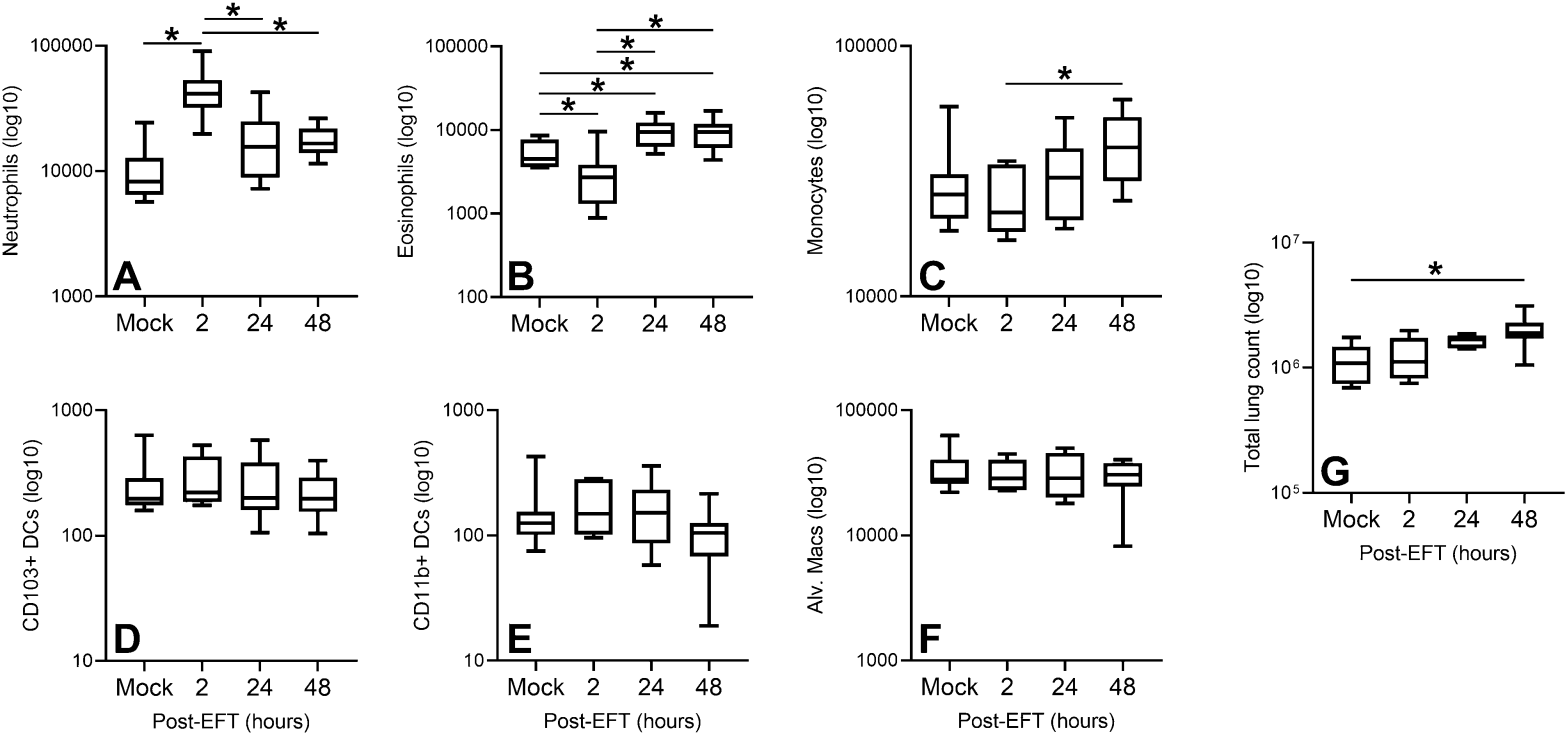
Lung immune cell infiltrates after EFT treatment in naïve mice. EFT (8 10 ms pulses at 200 V/cm) or mock control was administered to naive mice and mice were sacrificed at 2, 24, and 48 h post-EFT for lung cellular analysis by flow cytometry. Cell counts per lung for (**A**) neutrophils, (**B**) eosinophils, (**C**) monocytes, (**D**) CD103 + dendritic cells, (**E**) CD11b + dendritic cells, and (**F**) alveolar macrophages are shown. G) Total lung cell counts. Data are from two independent experiments, n = 7–10 for each group at each time point. Boxes are median line with first and third quartiles, min/max shown with brackets. *p < 0.05 by ANOVA.

As a first test of whether EFT associated with acute effects on immune cell infiltrates in the airways, lung tissue was taken at 2, 24, and 48 h after a single dose of chest wall EFT and flow cytometry performed on digested lung homogenates (gating strategy in Supp. Figure 1). Two hours after EFT, there was a transient increase in lung neutrophils that returned to control levels by 24 h post-EFT (Fig. 1A). Eosinophil recovery decreased at 2 h and then increased at 24 and 48 h post-EFT (Fig. 1B). Monocytes increased from 2 to 24 h post-EFT (Fig. 1C). No differences in dendritic cells and alveolar macrophages were observed over the time points (Fig. 1D–F). Overall lung cell recovery increased by 48 h post-EFT (Fig. 1G). Immunohistochemistry was performed on lung sections from mock and 2 h post-EFT groups to determine localization of neutrophils in the tissue. There was no difference observed in neutrophil localization within the parenchyma between mock and EFT treated samples, although a comprehensive analysis was difficult due to very low neutrophil numbers present in any given tissue section (data not shown).

**Figure 2:**
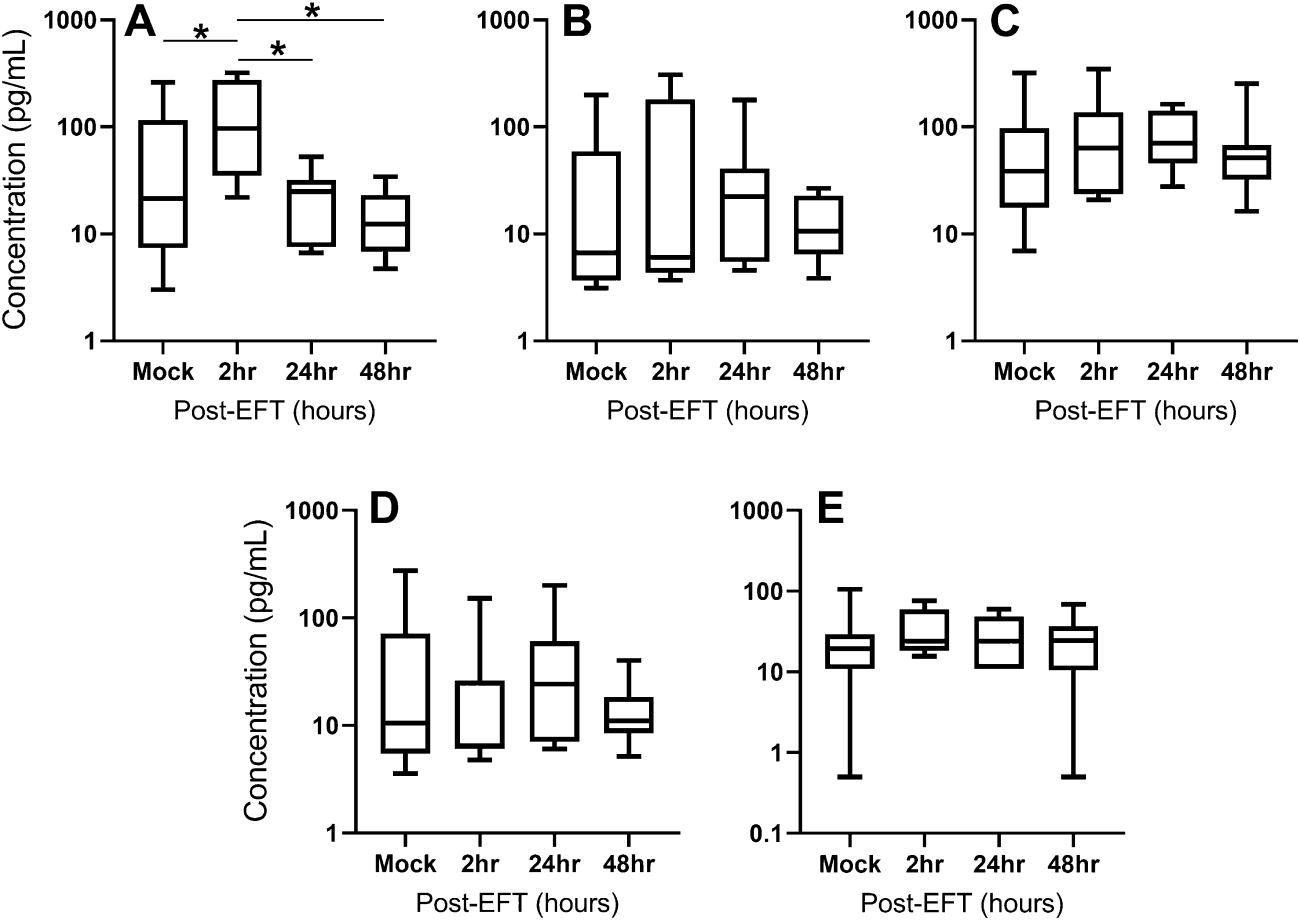
Inflammatory cytokine analysis of EFT and mock-treated naïve mice. After treatment as in Fig. 1, cytokines were measured in lung homogenates at each time point by bead-based multiplex. Cytokine levels for (**A**) IL-6, (**B**) IL-10, (**C**) IL-1β, (**D**) IFN-γ, and (**E**) GM-CSF are shown. Data are n = 7–13 for each group at each time point. Boxes are median line with first and third quartiles, min/max shown with brackets. *p < 0.05 by ANOVA.

Given the acute changes observed in cellular dynamics after EFT, bead-based multiplex assays were performed on lung supernatants to determine whether cytokine levels were altered by EFT. IL-6 levels increased at 2 h post-EFT and then returned to pre-EFT levels by 24 h (Fig. 2A). Levels of GM-CSF, IFN-γ, IL-1β, and IL-10 remained similar throughout (Fig. 2B–E). In order to determine whether the influx of neutrophils 2 h post-EFT may contribute to the increase in IL-6, neutrophils from Mock and 2 h post-EFT mice were enriched from enzyme-digested lung and cultured in the presence or absence of R848, an inducer of IL-6 in neutrophils^13^. IL-6 recovery from neutrophil cultures were comparable between groups in both the absence (Mock 174 +/− 93 vs. EFT 220 +/− 110 ng/ml, *p* > 0.05) and presence of R848 (Mock 279 +/− 85 vs. EFT 314 +/− 59 pg/ml, *p* > 0.05).

**Figure 3:**
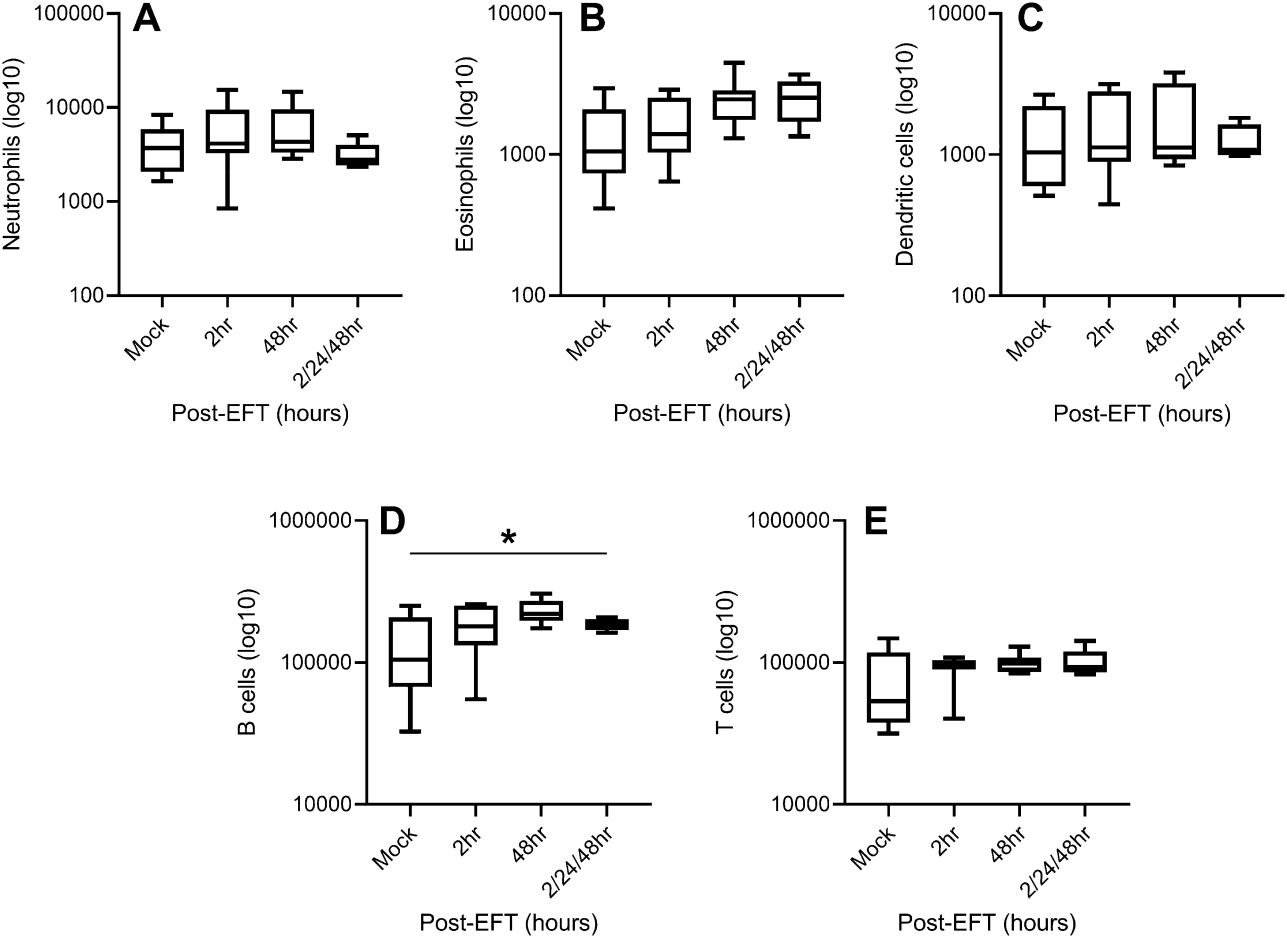
Analysis of spleen immune cells after EFT. EFT was administered in a single dose and mice sacrificed 2 and 48 h afterward, or EFT was given three times at 48, 24, and 2 h before sacrifice. Spleen cellular analysis by flow cytometry for (**A**) neutrophils, (**B**) eosinophils, (**C**) dendritic cells, (**D**) B lymphocytes, and (**E**) T lymphocytes is shown. Data are n = 5–8 for each group at each time point. Boxes are median line with first and third quartiles, min/max shown with brackets. *p < 0.05 by ANOVA.

A separate experiment was performed to determine whether EFT across the chest wall associated with changes in peripheral immune cell infiltrates. To test this, lungs and spleens were removed after chest wall EFT and flow cytometry performed. In order to determine whether repeat EFT applications had an additive effect, a group was included that received EFT at 2, 24, and 48 h before sacrifice. Lung cell recovery was similar to previous studies (Fig. 1 and data not shown). EFT induced minimal changes in spleen cellular infiltrates compared to control (Fig. 3). Three consecutive doses of EFT resulted in similar immune cell recovery compared to other groups, with only B lymphocyte recovery increasing versus control (Fig. 3E). These data suggest chest wall EFT may induce transient changes in local immune cell infiltrates that are not reflected systemically, and that EFT timing and dosing can be altered without dramatically changing immune cell recovery in lymphoid organs.

**Figure 4:**
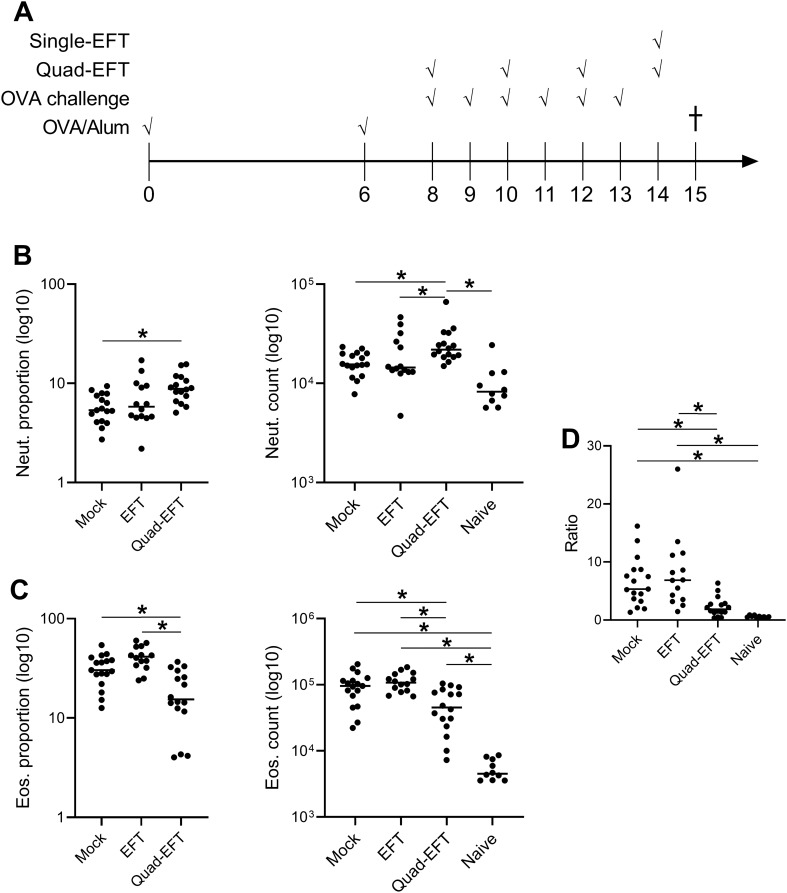
EFT-induced changes in lung immune cell infiltrates in a model of allergic airways inflammation. (**A**) Experiment time line. All mice except naïve were sensitized with OVA in aluminum hydroxide by intraperitoneal injection and then challenged with OVA by intranasal administration for 6 consecutive days starting at day 8. EFT was given as a single dose at day 14, or in four doses at days 8, 10, 12, and 14 (Quad-EFT) immediately prior to OVA challenge. Mock was performed at the same time points as Quad-EFT. Endpoint analyses occurred on day 15. Lung (**B**) neutrophil and (**C**) eosinophil proportion of total CD45 + cells (left graphs) and counts (right graphs) and (**D**) eosinophil:neutrophil ratio were determined by flow cytometry across 3 independent experiments, n = 14–17 per group. Lines show group median with individual mice shown as points. *p < 0.05 by ANOVA.

Since acute changes in eosinophil to neutrophil ratio were observed in the lung after EFT, a mouse model of Th2-mediated airway inflammation was utilized to determine if EFT associated with changes in lung cellular infiltrates after antigenic challenge. To this end, mice were sensitized with OVA in Alum and then challenged by intranasal route with OVA in the presence or absence of EFT (Fig. 4A). Different variations in the EFT approach were compared. First, we compared delivery of EFT across the chest wall (using pad electrodes) versus across the nasal bridge (using tweezer electrodes). Second, a single EFT dose versus four doses delivered every other challenge day (Quad-EFT) to the chest wall were compared (see cartoon, Fig. 4A). In all cases, EFT was delivered to animals immediately prior to OVA challenge. The single EFT group was given EFT on day 14, the same time point as the final treatment for the Quad-EFT group. A single EFT dose delivered across the nasal bridge was associated with minimal changes in lung immune cell infiltrates, with a slight reduction in neutrophils as the only change observed after OVA challenge (Supp. Figure 2 and data not shown). Multiple doses of nasal EFT was not done. However, mice receiving Quad-EFT across the chest wall showed a significant increase in neutrophils and decrease in eosinophils in lung compared to both mock and single EFT groups (Fig. 4B, C). Eosinophil recovery in the Quad-EFT group did not reach the levels seen in naïve, non-allergic mice (Fig. 4C). This resulted in a significant decrease in eosinophil:neutrophil ratio in the Quad-EFT group compared to mock control and single EFT mice, and comparable levels to that seen in naïve, non-allergic mice (Fig. 4D).

**Figure 5:**
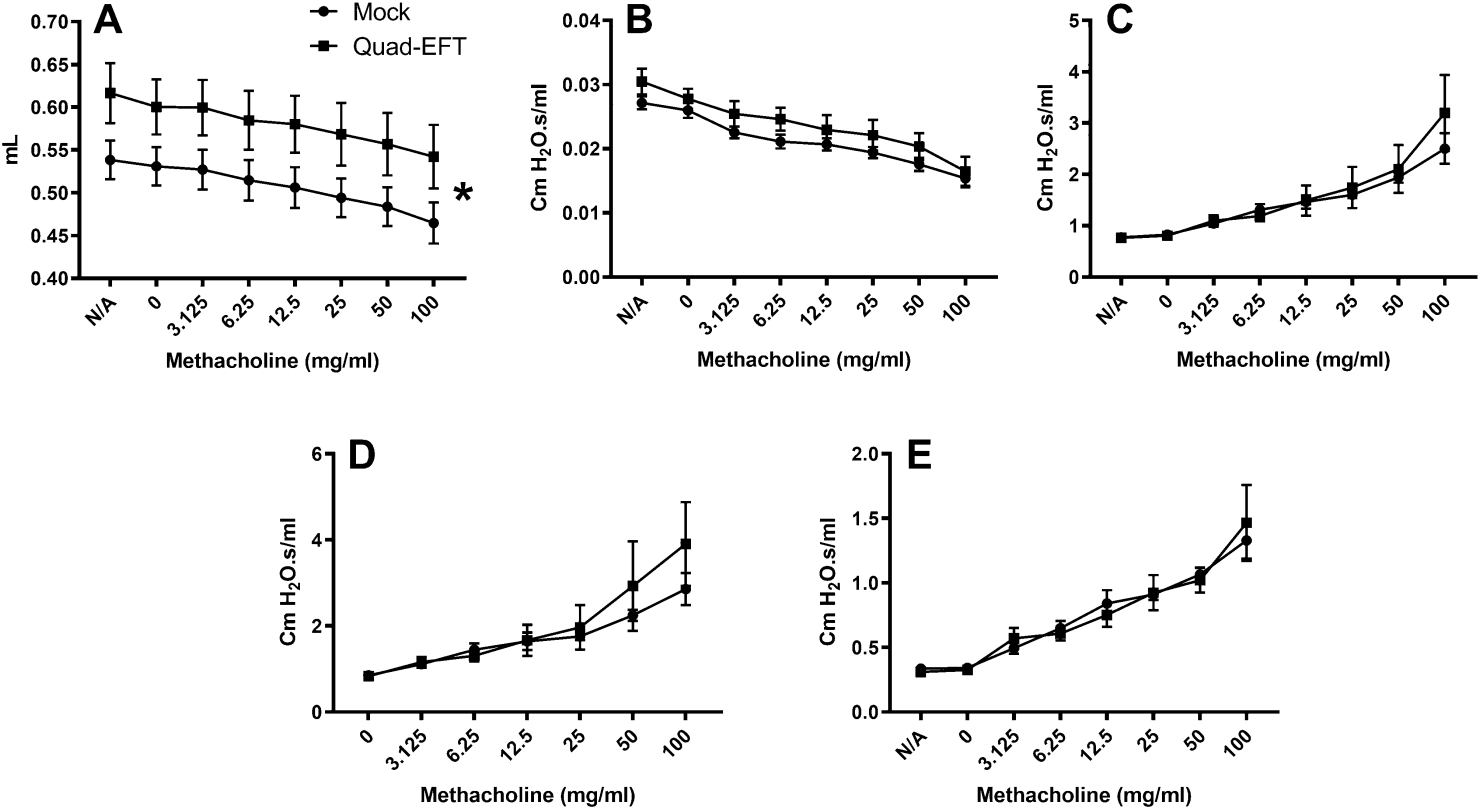
Lung function testing in EFT and mock-treated mice. Mice were subjected to the allergic inflammation model as in Fig. 4. Quad-EFT and Mock control groups were analyzed for lung function parameters using a flexiVent system at day 15. (**A**) Inspiratory capacity, respiratory system (**B**) compliance, (**C**) resistance, (**D**) maximum resistance, and (**E**) Newtonian resistance are shown. Data are shown as + / − SEM of n = 7–8 per group. *p < 0.05 by ANOVA.

In order to determine whether changes in immune cell infiltrates in the allergic inflammation model were associated with chemokine secretion, lung supernatants were tested by bead-based multiplex for chemokines known to direct leukocyte migration during inflammation. Although 8 of 9 chemokines tested had a lower mean recovery in the Quad-EFT group compared to control, none reached statistical significance combining three independent experiments (Supp. Figure 3). To determine whether EFT caused changes in lung function, OVA/Alum sensitized and OVA-challenged mice were subjected to flexiVent. Across the methacholine dose range used, inspiratory capacity was higher in Quad-EFT mice compared to Mock (Fig. 5A). Other measures of lung function were the same between groups (Fig. 5B–E).

## Discussion

These data investigate the application of EFT as a potential therapeutic strategy for modifying immune cell dynamics in the lung. The EFT dose chosen is within an acceptable range for exogenous electric field treatments already in use in humans^14^. Overall, EFT was associated with mild and transient changes in lung cells and soluble mediators, suggesting low toxicity to the tissue. Peripheral immune cells in the spleen were not as affected by EFT. Indeed, three consecutive EFT doses within 48 h in naïve mice did not result in any appreciable change in immune cell populations in our hands. While a wide EFT dose range was not tested in these studies, electric field dose may be tuned in order to focus its effects on specific cell types within a tissue. For example, since larger cells are permeabilized at lower field strengths than smaller cells, it is possible to electroporate specific cells within a tissue if they are sufficiently different in size than other cells within the tissue^4^. Therefore, refining dose and timing regimens may result in more targeted outcomes that avoid toxicity and optimize changes in cellular dynamics.

The most consistent finding was an increase in lung neutrophils across multiple experiments, which was surprisingly accompanied by a decrease in eosinophils after EFT. This observation was replicated using a model of lung allergic inflammation. The role of eosinophils and neutrophils in asthma is complex, with specific asthma endotypes often aligning with enrichment of eosinophils, neutrophils, or both. Neutrophilic or mixed phenotype asthma can be associated with steroid resistance, while eosinophilic asthma is often allergic in nature with highly polarized Th2-type inflammation^12,15^. Since EFT was able to alter the eosinophil:neutrophil ratio in the lung both acutely in naïve mice (within 2 h) as well as days after EFT in allergen-challenged mice, it is intriguing to explore how modification of these two critical cell types could alter clinical outcomes. The spike in neutrophils was accompanied by an increase in IL-6, which has been shown to be associated with asthma in humans^16–18^. IL-6 may be enriched in neutrophilic asthma^19^, and recent animal studies showed IL-6-deficient mice had increased lung-infiltrating eosinophils in an allergic asthma model^20^. This suggests that transient EFT-induced IL-6 production may be responsible for changes in cell accumulation to the lung. However, EFT-induced changes in neutrophils were also observed after OVA challenge when four doses were given, suggesting a longer-term effect may be possible if dose and timing are optimized. Further work is needed to understand the short and long-term impact of EFT on lung cellular infiltrates, and clinical phenotypes of asthma.

In order to explore EFT as a potential intervention in lung, it is critical to understand how EFT associates with lung function. Overall, there was minimal impact of EFT on lung function, which is encouraging from a therapeutic perspective. In our experiments, inspiratory capacity was the only parameter that showed a change with EFT. An increase in inspiratory capacity can occur after bronchodilator and/or combination treatments in patients with COPD or asthma/COPD overlap and is associated with increased lung function^21–23^. However, it is less clear as to the role of inspiratory capacity in asthma^24^. On a related note, lung compliance trended higher in EFT-treated mice but was not statistically significant. While these results are overall promising, more work is needed to determine if EFT can be a viable therapeutic approach to treat asthma or other inflammatory airway diseases.

## Supplementary Information

Supplementary Figures.

## Data Availability

The datasets generated during and/or analyzed during the current study are available from the corresponding author on reasonable request.
