# Supplementary material for: Changes in lung immune cell infiltrates after electric field treatment in mice
Source: Sci Rep. 2021 Jan 14;11:1453. doi: 10.1038/s41598-021-81174-y (PMC7809414; doi:10.1038/s41598-021-81174-y)

Data supplement for:

Changes in lung immune cell infiltrates after electric field treatment in mice

Sophia I Eliseeva, Zackery A Knowlden, Gillian M Schiralli Lester, David A Dean, Steve N Georas, Timothy J Chapman

Supplemental Figure 1: Flow cytometry-based identification of immune cell types. A) Lung cell identification. After identification of live, CD45+ single cells, population was split between Siglec-F+ and Siglec-F-. Neutrophils: Siglec-F-CD11b+Ly6G+. CD103+ dendritic cells: Siglec-F-CD11b-CD103+CD11c+MHC class II+. CD11b+ dendritic cells: Siglec-F-CD11b+CD11c+MHC class II+CD64-. Monocytes: Siglec-F-CD11b+CD11c-MHC classII-F4/80+. Eosinophils: Siglec-F+CD11b+Ly6G-CD11c-. Alveolar macrophages: Siglec-F+CD11b-CD11c+F4/80+. B) Spleen cell identification. After identification of live, CD45+ single cells, the following populations were identified: dendritic cells: CD11c+. Neutrophils: CD11b+Ly6G+. Eosinphils: CD11b+Ly6G-Siglec-F+. T lymphocytes: CD11b-CD11c-B220-. B lymphocytes: CD11b-CD11c-B220+.

**A**


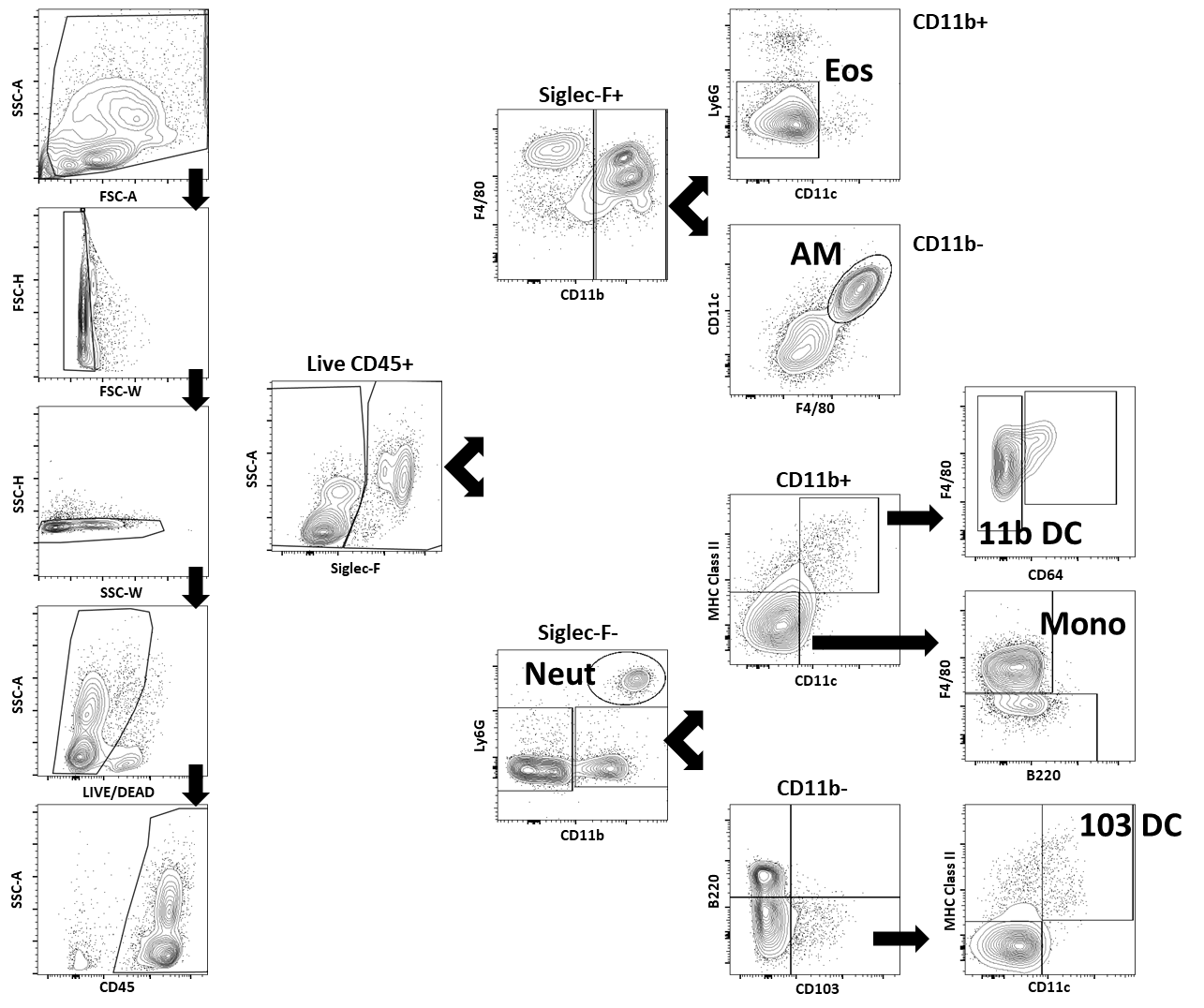


**
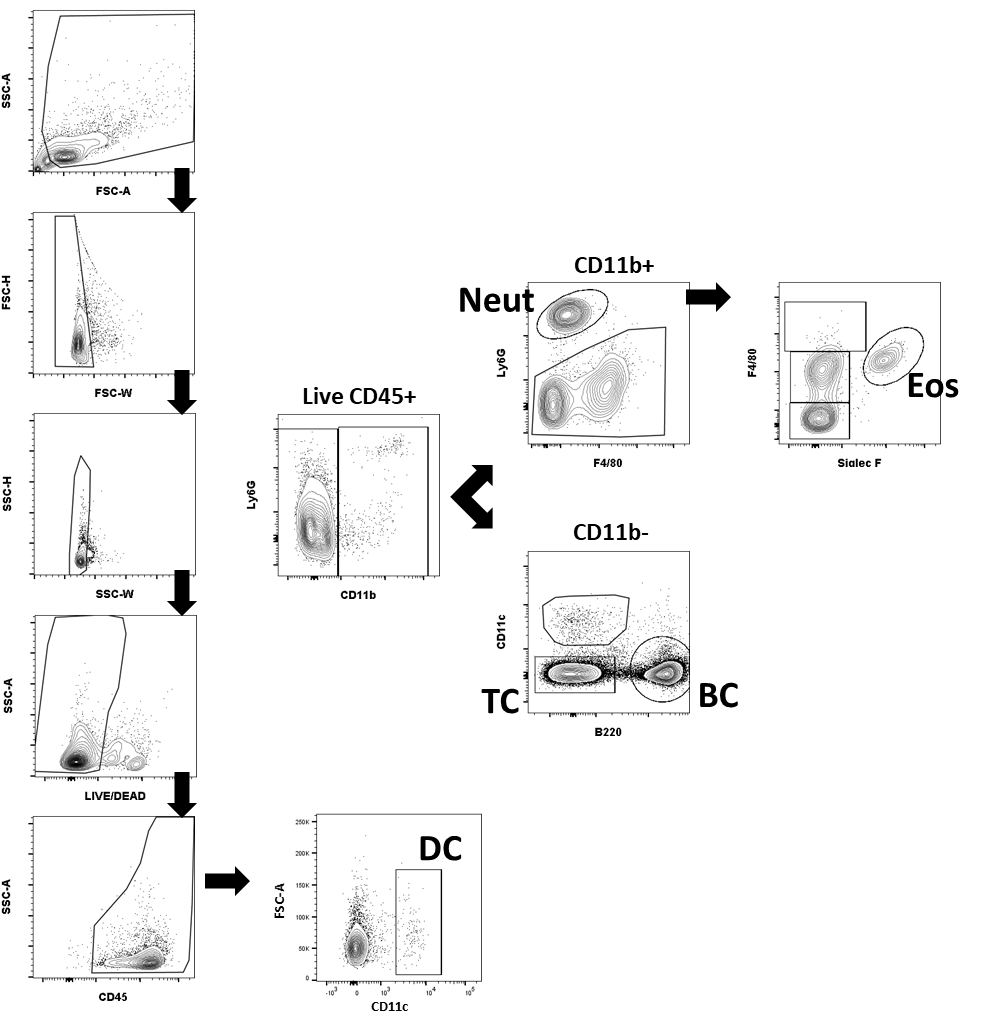
B**

Supplemental Figure 2: Comparison of nasal versus chest application of EFT in the allergic inflammation model. Mice were sensitized and challenged as in Figure 4, and EFT delivered across the chest wall or the nasal bridge on experimental day 14. On day 15, lung cell recovery of A) neutrophils and B) eosinophils was determined by flow cytometry. Data are from two independent experiments, n=12-14 per group. * = p<0.05 by Mann-Whitney test.


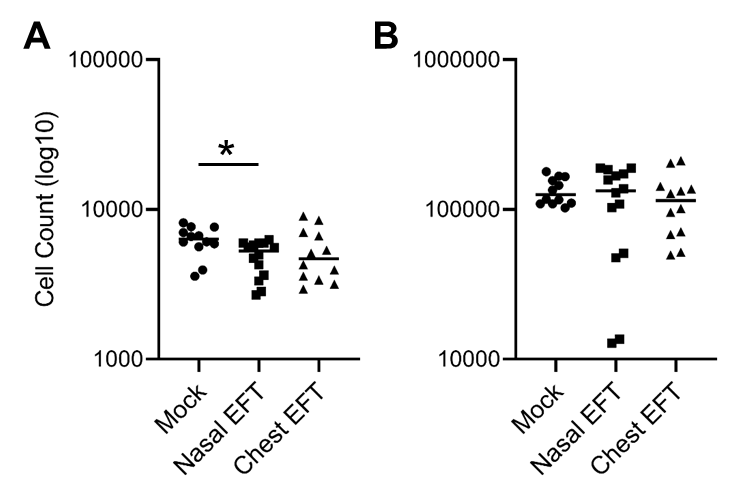


Supplemental Figure 3: Cytokine analysis of lung homogenates after Quad-EFT or Mock control. Lung homogenates from mice analyzed in Figure 4 were subjected to bead-based multiplex. Specific analytes are shown on the x-axis. Data are n=17 per group with 95% confidence interval shown.


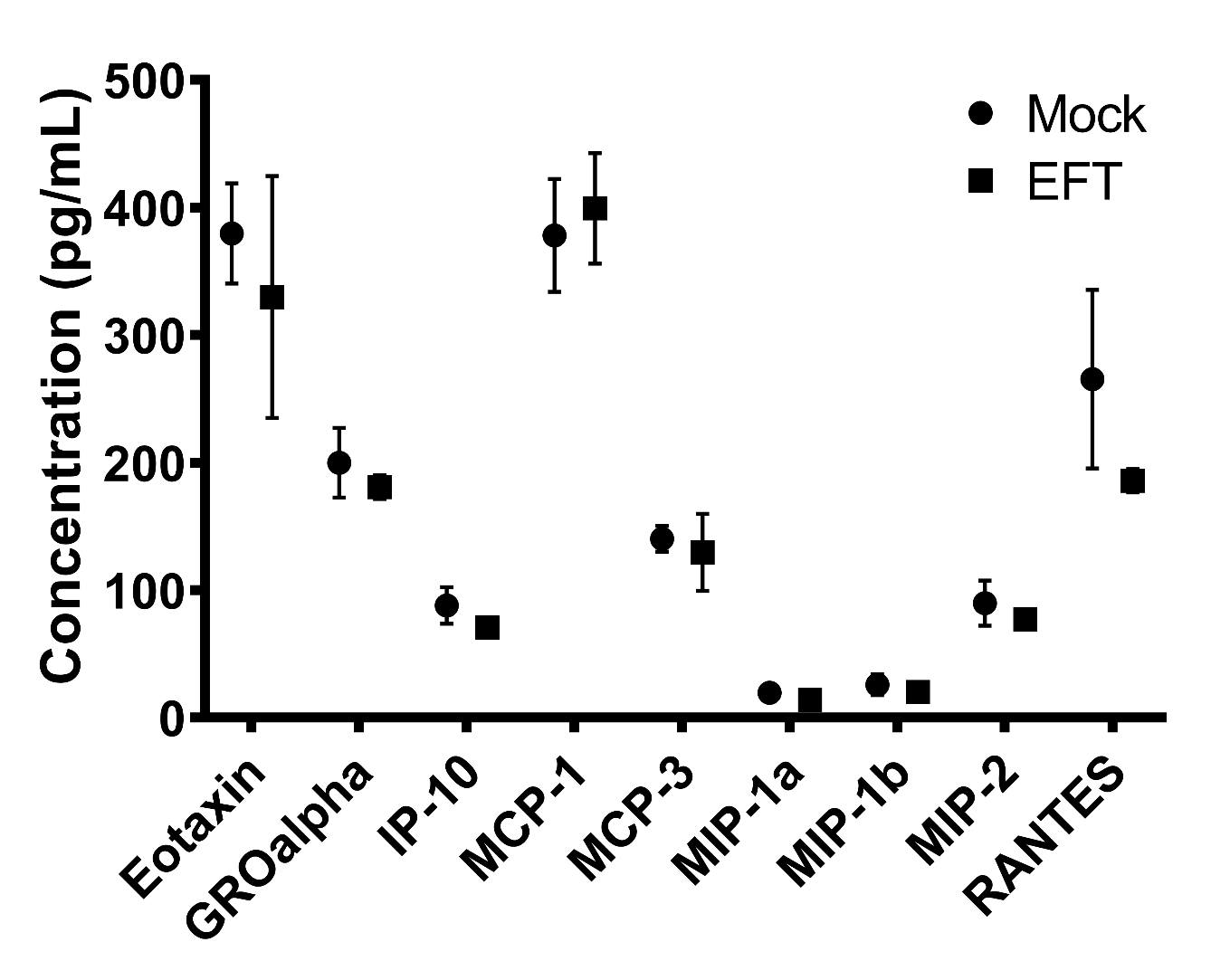

Supplement: Supplementary file 1 — Supplementary Figures. [file 41598_2021_81174_MOESM1_ESM.docx]
